# Influencing Factors on Postmortem Protein Degradation for PMI Estimation: A Systematic Review

**DOI:** 10.3390/diagnostics11071146

**Published:** 2021-06-23

**Authors:** Angela Zissler, Walter Stoiber, Janine Geissenberger, Peter Steinbacher, Fabio C. Monticelli, Stefan Pittner

**Affiliations:** 1 Department of Biosciences, University of Salzburg, 5020 Salzburg, Austria; angela.zissler@sbg.ac.at (A.Z.); walter.stoiber@sbg.ac.at (W.S.); janine.geissenberger@stud.sbg.ac.at (J.G.); peter.steinbacher@sbg.ac.at (P.S.); 2 Department of Forensic Medicine, University of Salzburg, 5020 Salzburg, Austria; fabio.monticelli@sbg.ac.at

**Keywords:** protein, degradation, postmortem interval, time since death, influencing factor, temperature, age, sex, body weight, insects

## Abstract

The present review provides an overview of the current research status on the effects of influencing factors on postmortem protein degradation used to estimate the PMI (postmortem interval). Focus was set on characteristics of internal and external influencing factors and the respective susceptibility and/or robustness of protein degradation. A systematic literature search up to December 2020 was conducted on the effect of influencing factors investigated in the context of postmortem protein degradation in the tissues of animals and humans using the scientific databases PubMed and Google Scholar, as well as the reference lists of eligible articles. We identified ten studies investigating a total of seven different influencing factors in degrading tissues/organs (*n* = 7) of humans and animals using six different methodological approaches. Although comparison of study outcomes was impeded by the high variety of investigated factors, and by high risk of bias appraisals, it was evident that the majority of the influencing factors concerned affected protein degradation, thus being able to modulate the precision of protein degradation-based PMI estimation. The results clearly highlight the need for a thorough screening for corresponding factors to enable the introduction of appropriate correction factors and exclusion criteria. This seems especially relevant for the protein degradation-based study of human PMI to increase the reliability and precision of the method and to facilitate a broader applicability in routine forensic casework.

## 1. Introduction

After death, a decomposing body undergoes complex unavoidable and irreversible biochemical, physical and physicochemical changes due to the lack of circulating oxygen, the cessation of anabolic production of metabolites, altered enzymatic reactions, and advancing cellular degradation [1]. Principally, postmortem changes occur in a regular fashion of successive degradation stages [2]. However, there are considerable variations due to a wide array of influencing factors resulting from both the individual body itself (intrinsic factors) and the environment (extrinsic factors). Intrinsic factors include body mass and surface area, but also age, sex, antemortem medical conditions, the presence of injuries/trauma, level of bacterial activity, and cause of death, among others [3,4,5]. Extrinsic factors include the presence and type of clothing and other insulation of the body, but also the environmental conditions at the death scene, in particular temperature, moisture, intensity of air flow, depth of burial, and activity of insects and microorganisms, etc. [2,3,5,6]. A thorough understanding of the susceptibility of postmortem changes to such factors is crucial, because these likely affect both the appearance of the changes as well as the rate of decay, thus either accelerating or decelerating the progression of postmortem events [7]. This is certainly one of the most relevant reasons why estimation of the PMI has remained a challenging task in forensic science for hundreds of years. Even though several ‘classical’ methods have been established to date [8,9,10], and new approaches are continuously being presented [11,12], all methods presently available turned out to have limitations that are often attributed to influencing factors. If unconsidered, this makes a method prone to error and inaccuracy, entailing limited practicability in routine forensic applications. Several proposed approaches for PMI estimation therefore remain in their early stages of development, and only rarely a has method progressed beyond the pilot stage and is actually involved in forensic casework such as the analysis of protein decomposition [13]. As outlined above, the recently demonstrated predictability of degradation patterns of proteins [14,15,16] is limited by effects of various influences [12]. A key to improve the method is to obtain a deeper understanding of the effects of intrinsic and extrinsic factors, enabling the specification of suitable correction factors and/or exclusion criteria that, in turn, enhance their reliability and applicability. With this systematic review, we aim to reveal and to summarize the current research status regarding the types of influencing factors acting upon postmortem protein degradation, and the susceptibility and/or robustness of the degradation processes to each of these factors in human and animal tissue.

## 2. Methods 

### 2.1. Data Source and Eligibility Criteria

An evidence-based systematic review of the literature was conducted according to the PRISMA guidelines to evaluate the current status of research focusing on the effect of influencing factors on postmortem protein degradation. A focused research question was defined using a PICo (Population, Interest, Context) framework, serving to assess: (i) which influencing factors have been investigated; and (ii) to what extent these factors affect postmortem protein degradation (I) in various tissues/organs of animals and humans (P) analyzed to estimate the PMI in forensic science (Co). The review aimed to include articles that: (i) directly investigated the effects of influencing factors of postmortem protein degradation in similar study settings; and/or (ii) articles that statistically evaluated such effects in a targeted cluster analysis.

The systematic search for eligible literature was performed using the electronic database PubMed. In addition, an extended electronical search in Google Scholar was performed to identify studies not listed in PubMed (e.g., studies published in open access journals). The search was complemented by a screening of the reference lists of the articles principally eligible for inclusion. A preselection of studies was conducted electronically based on the following predefined inclusion criteria: “peer revision”, “English language”, “availability of full text” and “publishing date between 2000 and 2020”. The timeframe was selected based on a previous study [15], a systematic review article on protein decomposition-based research in the forensic context, in which it was shown that the earliest relevant research originated in 1999.

### 2.2. Search Strategy

The systematic search was conducted using different Boolean operators. The key words included predefined influencing factors known to affect postmortem body changes. The following search queries were used: (1) “(temperature OR ADD OR accumulated degree days)”; (2) “(humidity OR moisture OR wind OR air flow OR weather OR season)”; (3) “(exposure OR burial OR submersion OR coverage OR containment OR clothing OR outdoor OR indoor)”; (4) “(disease OR injury OR medication OR intoxication OR therapy)”; (5) “age”; (6) “(body mass OR BMI OR weight)”; (7) “(sex OR gender)”; (8) “(cause of death OR circumstances of death)”; and (9) “(bacteria OR microorganisms OR insects)”, each combined with: “AND (protein degradation OR protein decomposition) AND postmortem”. Google Scholar searches were conducted with the abovementioned keywords and Boolean operators, also combined with the terms “post mortem interval” and “time of death” and “time since death”. The last date of search was 14 December 2020. The process was completed by a hand search for references cited in the identified studies.

### 2.3. Study Selection

In accordance with the PRISMA guidelines, identified records were independently evaluated by two of the authors. After the removal of duplicates, titles and abstracts were screened and records not relevant to the review were systematically excluded using the following predefined filters:

**filter I**

- Study content other than forensic;
- Type of article other than original research article, review (meta-analysis), or case study;
- Type of research target other than tissue/organ;
- Irrelevant articles not excluded by electronical preselection (e.g., other language, no full text).

**filter II**

- Biomarker other than proteins;
- Aim other than estimation of the PMI;
- No investigation of influencing factors.

All articles of appropriate forms (i.e., original research articles, review articles/meta-analyses and case studies) reporting on influencing factors of postmortem protein degradation in animal and/or human tissues in a forensically relevant context were included, and full texts were further screened for final eligibility. Discrepancies between the two evaluating authors regarding eligibility were discussed until a consensus was reached.

### 2.4. Risk of Bias Assessment

The risk of bias of individual studies was assessed using a previously published framework ([15]), which we also deemed appropriate for use in the present review (File S1, File S2). Based on the Cochrane Risk of Bias tool RoB 2.0 [17], this established matrix involves specific signaling questions summarized in six domains, including general study design, precision in reporting, presence of outcome data, outcome measurements, selective outcome reporting, and the multiple use of data. Risk of bias assessment for each included study was performed independently by two of the review authors. Disagreements were resolved by discussion between these authors. Judgements were expressed as low, moderate and high risk of bias, again in agreement with the recommendations of the Cochrane Risk of Bias collaboration [17].

### 2.5. Data Extraction and Synthesis

Due to the small number of identified studies and their heterogeneity in relation to experimental design and concomitant fundamental differences in outcome measurements and results, data could not be combined for meta-analysis, but were instead synthesized in a descriptive approach. For this purpose, two authors independently extracted the following study characteristics using standardized data extraction forms: (i) general study characteristics (including author and year of publication, study type, investigated influencing factor, research target and method, sampling size and study groups); and (ii) specific study details (such as storing conditions, investigated PMI, analyzed proteins, information on measurement procedures and outcome, etc.). Extracted data were-cross checked for accuracy.

Studies were grouped depending on the influencing factors investigated, and compared in terms of study design, outcome and established risk of bias. Due to the small number of eligible studies investigating similar influencing factors and high risk of bias classifications, no complete appraisal of evidence across included studies could be carried out (File S3). Nevertheless, the authors commented and discussed the evidence base, consistency of the results, and the generalizability of outcomes wherever possible.

## 3. Results

### 3.1. Study Selection

**Figure 1:**
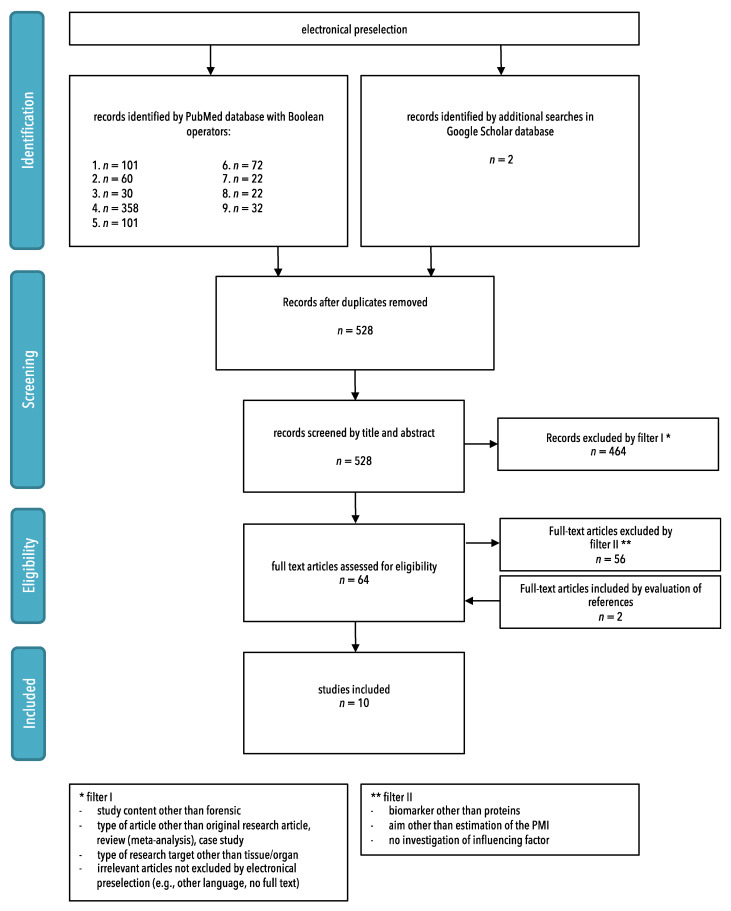
Flow chart of the systematic literature search according to PRISMA guidelines.

The systematic search in the PubMed database yielded a total of 798 records (Figure 1). The expanded searches in Google Scholar revealed two additional studies with relevant content. A total of 800 studies were searched for duplicates, resulting in the removal of 272 items. Screening of titles and abstracts of the remaining articles led to the removal of another 520 articles which failed to pass the predefined eligibility filters. Most of the studies omitted in this step lacked a relationship with forensic science, instead dealing with topics such as meat science or clinical matters. This being completed, eight full text articles remained which met all inclusion criteria set for this study. Screening of the literature references cited in these articles resulted in the identification of two further valid studies, thus giving a total of 10 studies appropriate for inclusion in this review.

### 3.2. Characteristics of Included Studies

**Figure 2:**
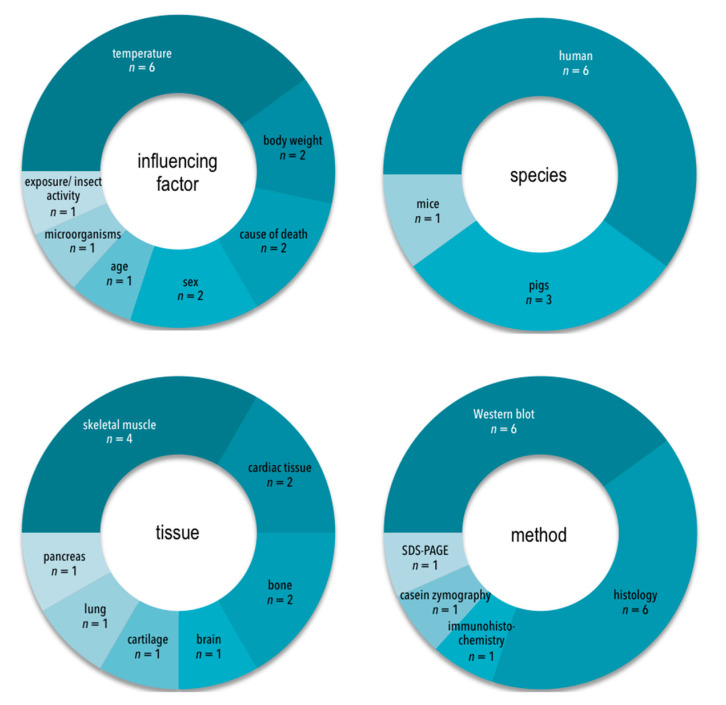
Frequency diagrams of analyzed influencing factors, species, tissues and methods.

All of the 10 eligible studies were original research papers. Seven different influencing factors were investigated. Most studies (8 out of 10) focused on the effect of one influencing factor; two studies investigated the effect of several factors. The most frequently analyzed influencing factor was temperature (*n* = 6), followed by body weight, cause of death and sex (*n* = 2 each). The effect of age, exposure and microorganisms was examined in a single study each. Six studies utilized human tissues; four studies used animal models (pigs *n* = 3, mice *n* = 1, Figure 2). Autopsy-derived human tissues were in some cases intermittently stored to enable multiple sampling. By contrast, three of the animal studies for this purpose relied on the storage of whole cadavers. The number of samples taken from individual corpses, and coincidently number of sampled individuals, varied broadly, ranging from 2 to 500. Most studies (8 of 10) investigated the effects of influencing factors in a single type of tissue; two studies examined two tissues. The most frequently analyzed tissue type was skeletal muscle (targeted in four studies), followed by cardiac tissue and bone (*n* = 2 each). Brain, cartilage, lung and pancreas tissues were each used in a single study (Figure 2). Seven different methodological approaches were used, either alone or in varying combinations: 6 of the 10 studies used Western blots, 6 of the 10 studies employed classical histology, combined with digital analysis (*n* = 2), or photometrical analysis of destaining solution (*n* = 2), or expert analysis by a grading scale (*n* = 1) (Figure 2). One study relied on immunohistochemistry. One study each used casein zymography and sodium dodecyl sulfate polyacrylamide gel electrophoresis (SDS-PAGE) as a secondary method.

Relevant characteristics of the individual studies, e.g., regarding storage conditions, PMI range, sampling procedures, investigated proteins, types of analyses exerted, and the main results, are presented in Table 1, assorted by investigated influencing factors. A more comprehensive summary of all individual studies, including the individual risk of bias appraisal, is provided in the Supplementary Materials (File S4).

### 3.3. Risk of Bias Assessment

**Figure 3:**
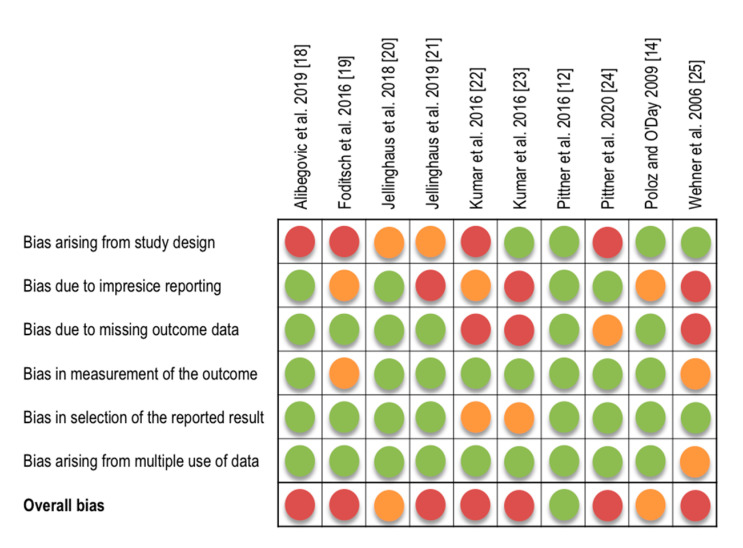
Risk of bias assessment of included studies. Green, low risk of bias; yellow, moderate risk of bias; red, high risk of bias.

Only one study was associated with a low risk of bias [12]; two studies had a moderate risk of bias [14,20]. The majority of studies (7 out of 10) were rated with an overall high risk of bias. This was due to several reasons, including: (i) the use of unfavorably small sample sizes of 1–3 individuals (e.g., one individual per tested temperature regime); (ii) imprecise or missing reporting on sampling procedures and sites, and procedures of measurement and data analysis, together impeding reproducibility; and (iii) lack of complete outcome data. Only one study [21] reported blinding of the assessors, even though the combination of subjective evaluation based on unclear criteria and lack of blinding is particularly prone to increase the risk of bias. This adds importance to insufficient information on immunostaining intensity classification in two of the studies [19,25]. Details of the risk of bias assessments for all included studies are presented in Figure 3 and in Table S1.

### 3.4. Body of Evidence

#### 3.4.1. Evidence Base

The risk of bias assessment resulted in high-risk appraisals for the majority of articles investigating the influence of temperature, leaving only a single level I and II study, with low and moderate risk of bias, respectively. The evidence base for the effect of temperature on protein degradation from included studies was therefore rated poor. This is the same for all other influencing factors investigated by identified studies (File S4).

#### 3.4.2. Consistency

Outcomes regarding the influence of temperature are mostly consistent across studies, but different study designs exacerbate a clear evaluation. From this study’s results, it seems plausible that effects of temperature are different across species and tissues (discussed in the following sections). Study designs for all other influencing factors tested were largely different, hindering comparisons across studies regarding consistency.

#### 3.4.3. Generalizability

In most studies (across various influencing factors) using human individuals, populations studied represented cases with characteristics typical for routine forensic practice. The evidence from human studies seems therefore principally generalizable for the target population.

## 4. Synthesis of Results

### 4.1. Temperature

**Figure 4:**
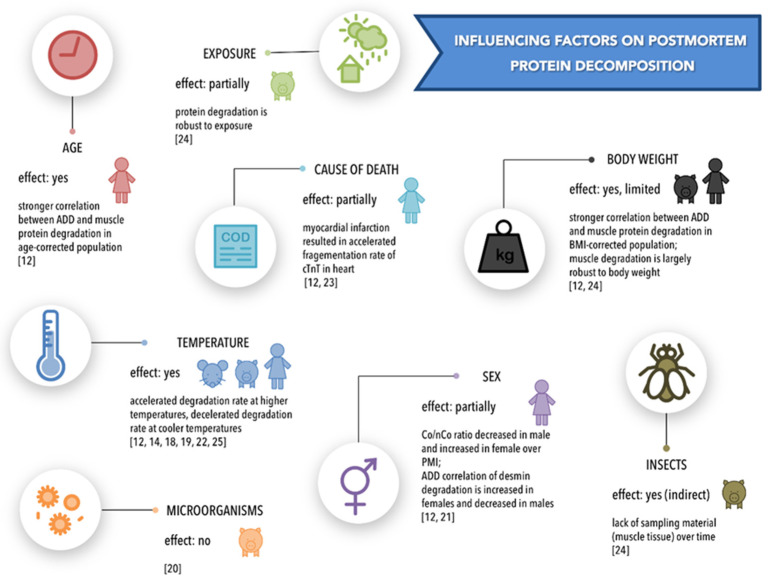
Effects of influencing factors [12,14,18,19,20,21,22,23,24,25].

Six of the ten studies investigated the effects of temperature on postmortem protein degradation. Temperatures investigated ranged from 4 °C through 5 °C, 10 °C, 12 °C, room temperature (21 °C and 22 °C), and 25 °C to 37 °C (Table 1). Analyzed tissues were brain, cartilage, cardiac tissue, lung, pancreas and skeletal muscles of humans and animals (mouse, pig), with PMIs ranging from 0 to 38 days. Methodological approaches included casein zymography, histology, immunohistochemistry, SDS-PAGE and Western blots. Four of the studies reported an increased degradation of proteins at higher temperatures, compared to lower temperatures [14,19,22,25] (Figure 4). In contrast, one study found no significant effect of temperature, instead indicating similar degradation rates in samples stored at low (11 °C) and high (35 °C) temperatures [18]. One study referred indirectly to temperature effects by the employment of accumulated degree days (ADD) [12], a measure of (thermal) energy accumulation required for the chemical and biological reactions occurring during decomposition [9]. In this particular study, the majority of protein degradation events were found to correlate with ADD, thus indicating complex coherences of postmortal time and temperature.

### 4.2. Body Weight

Two studies investigated the effect of body weight on skeletal muscle degradation using Western blots. The body mass of human corpses was defined in terms of body mass index (BMI) [12], and that of pig cadavers by body weight (in kg) [24]. Results from 40 human cases support a BMI-related effect, showing that the correlation between protein degradation and ADD is stronger when evaluation is confined to a BMI-corrected group in which individuals with a BMI below 19 and above 30 were excluded [12] (Figure 4). In a field study using pig cadavers, the degradation of skeletal muscle proteins was found to be largely robust against the factor “body weight” [24].

### 4.3. Cause of Death

The effect of cause of death on protein degradation was investigated by two Western blot-based studies using human skeletal muscle [12] and cardiac tissue [23]. Using a targeted cluster analysis, one of these studies [12] evaluated possible effects of internal malfunction and organ failure (27 cases), external trauma (10 cases), intoxication (one case), and unknown cause (2 cases), and found no effect of the cause of death on protein degradation dynamics. The second study [23] compared protein degradation in cases with a different set of causes of death, comprising asphyxia, poisoning, burn, electrocution, and myocardial infarction, supplemented by controls (cause of death not defined). Results document that the degradation of intact cardiac troponin T (cTnT) was fastest in the myocardial infarction group (Figure 4).

### 4.4. Sex

Two studies evaluated the effect of sex on protein degradation in human skeletal muscle [12] and bone [21] using targeted cluster analysis after a demonstration of postmortem protein degradation with Western blots and a stereoscopic analysis of histologic specimens, respectively. For skeletal muscle proteins, the results revealed no major differences between male and female sex, with one exception: skeletal muscle desmin exhibited a sex-specific degradation pattern, with a slightly faster decomposition in women (Figure 4). For bone protein decay, concentrations of histologically stained collagen in males were demonstrated to decrease with advancing time postmortem, whereas an unexpected increase in the Co/NCo ratio with progressing PMI was found in females.

### 4.5. Age

Only one of the included studies [12] investigated the effect of age on postmortal protein degradation in human skeletal muscle, using a targeted cluster analysis and ADD. It was found that the correlation between protein degradation and ADD increased if assessed in an age-corrected group, indicating that degradation rates in the central group of the age distribution (18 to 80 years) diverged from those in the peripheral age groups (below 18 years and above 80 years) (Figure 4).

### 4.6. Exposure/Environment and Insect Activity

Again, only one study investigated the effects of exposure conditions (e.g., in the shade, in full sunlight, under a covering of branches) on skeletal muscle decomposition in pig cadavers, which were found to be largely robust against influences of exposure, although the effect was not evaluated at the protein degradation level [24]. In addition, progressing decomposition, and particularly increasing insect activity, impeded muscle tissue sampling after a PMI of only one week (Figure 4).

### 4.7. Microorganisms

Another single study investigated the influence of microorganisms on collagen degradation in bones [20]. For this purpose, boxes with buried bones were flooded with hay infusions and compared to a control group (distilled water). Stained histological sections enabled microscopic evaluations of Co/NCo ratio changes (for methodological details see [26] and File S4). The authors found a reduction in Co/NCo ratios over the investigated 3-month period, but no significant effect of the presence of microorganisms (Figure 4).

## 5. Discussion

By identifying 10 eligible articles, the present systematic review reveals substantial grounds for including a set of influencing factors as relevant determining parameters into the recent approach of using protein degradation in forensic PMI estimation. All except for one of the influencing factors were found to affect protein degradation dynamics in the respective study settings. This highlights the need to establish appropriate correction factors and/or exclusion criteria to increase the validity and precision of the protein degradation-based PMI estimation method. At the same time, the present review clearly indicates the need for further high-quality research in this field. A large part of the included studies, although often well designed, are of pilot character, mostly due to small sample sizes. This and other limitations often led to a high risk of bias, impeding the appraisal of the respective evidence. Results available to date are additionally relativized by inconsistencies in the number and combination of investigated factors, study design and methodology, and non-standardized choices of tissues. Thus, the following discussion does not stand on a solid evidence base (cf. body of evidence), but should rather provide indications and incitation for future research in this field.

### 5.1. The Effect of Temperature

It was already plausible from the observations of Wehner et al. [25] that protein decomposition is faster in the warmer months of the year than in the winter months [25]. This is in line with findings from animal decomposition under standardized conditions, demonstrating a statistically significant effect of storage temperature on protein degradation, with the fastest breakdown rates at the highest temperatures, and delayed degradation under cool conditions [14]. These findings have been repeatedly confirmed in other research fields (e.g., meat science [27,28,29]). However, to establish corrective factors that are applicable to temperature effects on protein degradation in routine cases inevitably requires further human decomposition studies, particularly because the temporal patterns of body cooling highly likely diverge between species [15]. Admittedly, it is comparatively easy to test temperature effects on protein degradation dynamics in animal models under standardized laboratory conditions, but extremely challenging to do the same in human tissues, for which exact temperature profiles between death and autopsy (sampling) are often unavailable. However, with certain constraints, also due to ethical reasons, researchers have already attempted to overcome this limitation by storing organs and/or pieces of human tissue under controlled/defined temperature conditions [18,22]. This has served to establish a fundamental temperature dependence of troponin T degradation in cardiac muscle [22], again showing the fastest breakdown at the highest temperatures in combination with a long PMI. A similar behavior has been reported for skeletal muscle and brain proteins [30,31]. Accelerated degradation at higher temperatures was also found for collagen and proteoglycans in cartilage, although in this case exceeded in importance by the influence of time [18]. This suggests that the effect of temperature on protein degradation differs between the various human organs/tissues (probably indicating a general divergence between “soft”/collagen-poor tissues and “hard”/collagen-rich tissues). However, this will remain partly speculative until additional high-quality studies that address this matter are available. Generally, in this context, it remains to be clarified whether the extent of temperature influences on postmortem protein degradation in an explanted tissue is comparable to that in an intact body. Additionally, special antemortem and perimortem conditions (e.g., diseases, drugs/medication) and specific causes of death (e.g., burns) with potential lasting effects on body core temperature [32] will have to be evaluated and taken into account when defining corrective factors and/or exclusion criteria.

An alternative approach to define and describe temperature effects on protein degradation in a standardized manner is to use accumulated degree days (ADD) as a reference [12], often briefly expressed in degree days (°d). This is particularly favorable in routine cases with varying environmental temperature conditions between death and sampling. The method has already been successfully employed in both animal models and humans to predict postmortem changes in corpse morphology [9] and insect colonization [33], and ADD have been significantly correlated with protein degradation [12]. Their use can easily be implemented; therefore, ADD may be recommended for further validation in future experimentation.

### 5.2. The Effect of Body Mass

Body mass, often expressed in terms of BMI, is a factor with multiple potential influences on postmortem protein degradation, firstly—but not exclusively—because mass affects postmortem thermal change in a reciprocally proportional manner [3]. Complying with this relationship, there is various evidence demonstrating that the BMI affects muscle protein degradation after death in humans [12] (Figure 4). For two proteins (cTnT and calpain), a nonlinear character of the relationship was indicated by the finding that the exclusion of individuals with a BMI below 19 and above 30 results in a stronger correlation between both degradation and ADD than in the uncorrected group. Remarkably, no similar effect was found for desmin [12]. In contrast, a study in pigs found that protein degradation was largely uninfluenced by the body weights of the cadavers [24]. However, because no statistical correlations were provided, the finding must be downgraded and conclusions about species-specific differences require further verification. The latter holds also for possible differences in the susceptibility to this factor between organs/organ parts, tissues and proteins. This seems to be relevant when comparing the degradation of proteins or their respective isoforms between upper leg muscles and lower leg muscles, or between striated skeletal muscle and striated cardiac muscle of corpses with mass-related different postmortem cooling behaviors. Further research is therefore required to clarify the apparently diversified influence of the factor “body mass” on protein degradation dynamics. The new research may again be preferentially undertaken in human studies than with animal models. Even if offering similar body mass conditions, species may exhibit different cooling behaviors due to different body proportions [34] indirectly affecting protein degradation rates.

### 5.3. The Effect of Sex and Age

The present analysis delivers a heterogeneous picture of how the factors sex and age exert influence on the postmortal degradation of proteins. In skeletal muscle, desmin turned out to be the only protein that exhibits differences between males and females, showing faster decomposition in women. The mechanism behind this difference is unclear, just as whether there is a relation to the fact that desmin is one of only a few muscle proteins with higher expression levels in men than in women [35]. A further speculative aspect relating to indirect influences is that male corpses cool more rapidly than female corpses of identical weight due to the higher content of body fat in the latter [5]. Further studies are required to clarify whether this indeed causes accelerated proteolytic degradation of proteins with special temperature-susceptibility.

A limited body of evidence also exists in relation to age effects on protein degradation postmortem. The unexpected increase in the Co/NCo ratio with progressing PMI in female human bones has been suggested to be connected to the high average age of the investigated female cases (majority above 65 years), with a possible association with age-related diseases such as osteoporosis [20]. In addition, an effect of age on protein degradation also seems to exist in skeletal muscle tissue [12], the correlation being stronger if very young and very old persons are excluded from the investigated cohort. This bears similarity and indicates a connection to thermal effects (see above). Additionally, it is indeed tempting to speculate that differences in thermoregulation of aged persons compared to middle-aged adults lead to deviations in postmortem cooling behavior [32]. Additionally, here, additional studies are required to further clarify these issues.

### 5.4. The Effect of Cause of Death

Relating to possible effects that could be exerted on postmortem protein degradation by variants of cause of death, there seems to be evidence that myocardial infarction affects postmortem troponin (cTnT) degradation in cardiac tissue [23]. Troponin is a common biomarker for cardiac injury; therefore, its degradation in myocardial samples has also been investigated in previous immunostaining-based studies. It was found that staining for cTnT and cardiac troponin I (cTnI) in human, canine, porcine, and rat heart muscle was absent or significantly decreased after myocardial infarction [36,37]. Similarly, a porcine heart failure model using Western blots showed an up to 70% reduction in cTnT and cTnI immunoreaction intensity at two months post-infarction [38]. In contrast, cTnT degradation was not influenced by the cause of death in skeletal muscle [12], nor was any other muscle protein tested. These contrasting findings support a heterogeneous susceptibility of individual protein isoforms to influences exerted by death-causing processes, and again highlight a need for extended further investigations.

### 5.5. The Effects of Exposure, Insects and Microorganisms

A cadaver is usually exposed to numerous factors potentially influencing protein decomposition behavior and rate [39,40]. Among these factors, conditions of surface exposure to the environment, and colonization by insects and microorganisms, are most plausible candidates for influencing postmortem protein degradation. This is, however, not fully sustained by the limited evidence from the studies included in this review. Thus, no (major) effects of exposure/environment and microorganisms on protein degradation in skeletal muscle and bones were found in pigs [20,24]. Modulatory effects of insect activity may be more substantial but difficult to examine in the long term, because increased insect activity has been found to impede muscle tissue sampling in the advanced PMI range. This demonstrates a restricting effect of insects (and other scavenging) on postmortem protein analysis in soft tissues. The implications remain largely unexplored.

## 6. Limitation

Due to the small number of identified studies, entailing that only one single article was available for most of the relevant influencing factors, and aggravated by the high degree of inhomogeneity among the included studies regarding design (species, PMI, etc.) and study outcome, no meta-analysis could be conducted. In addition, although extensive effort was made to identify all relevant work presently available, it is possible that some studies of relevance have been missed because of non-availability in the searched databases, or incompatible terminologies or language (i.e., only research articles in English could be considered).

## 7. Conclusions and Future Work

In the field of forensic PMI estimation, constant improvements of the understanding of all kinds of postmortal changes and their susceptibility to various influencing factors must be a prime goal. This is particularly relevant and urgent for analyses at the molecular level. The set of direct conclusions that can be drawn from the present literature analysis is that: (i) it confirms that a variety of factors are able to influence/modulate postmortem protein degradation; (ii) it highlights a largely unexplored complexity of the matter; and (iii) extensive further research and standardization is required to finally establish and fine-tune protein decomposition analysis as a most promising tool of forensic PMI determination. Animal models can certainly contribute to achieve this, mainly to substantiate and extend the basics of the method, including an assessment of effects of influencing factors such as temperature (cf. [14,19]). Efficient use may therefore be made of the advantages of animal models, e.g., the availability of controls, standardized physico-chemical conditions, appropriate sample size, limited costs, and no previous medical history. However, the results of the present work also indicate that caution must be taken when extrapolating data from animals to humans [15,41]. Particularly when aiming to establish correction factors and/or exclusion criteria to compensate for secondary factor effects, it seems inevitable that examinations should be performed in the human system. Only data from human studies with appropriate sample sizes, at best also allowing for multi-sampling of corpses stored at controlled environments, may suffice to produce data supporting accurate mathematical degradation models that can be used to estimate the PMI in routine cases, often with only a partly reconstructable history. To achieve this will likely also require the inclusion of further antemortem and postmortem influencing factors. Physical condition/state of training, disease, intoxication, etc., just as soil composition/chemistry at site of burial, circumstances of submersion, etc., should be considered whenever postmortem protein degradation is used for PMI estimation.

## Figures and Tables

**Table 1:** Study characteristics, details and main outcomes. PMI, postmortem interval; hpm, hours postmortem; dpm, days postmortem; pm, postmortem.

General Study Characteristics					Study Details and Outcome							
Author/ Year	Influencing Factor	Research Target (Species, Tissue)	Sample Size and Study Groups	Method	Storage Conditions	Investigated PMI	Sampling Site Details	Sample Number and Sampling Frequency	Investigated Proteins	Type of Measurement Procedure/ Data Processing	Type of Study Outcome	Main Study Outcome
Alibegovic et al., 2019 [18]	temperature	human, cartilage	3 individuals	histology/ grading scale	varying before autopsy; laboratory-controlled after autopsy, storage of samples in tubes, 11 ± 2 °C, 35 ± 2 °C	estimated PMI (30-48 hpm) + 1–36 dpm	human trochlea, medial and lateral condyle	3 samples per time point (3) and per temperature (2)	collagen, proteoglycan	qualitative assessment of histological staining intensity using Bern grading scale	significant decrease in staining intensity over PMI	no significant effect of temperature
Foditsch et al., 2016 [19]	temperature	pig, skeletal muscle	2 individuals (1 per group)	SDS PAGE, Western blot	4 ± 1 °C, 22 ± 2 °C	4 °C: 0–21 dpm, 22 °C: 0–5 dpm	M. biceps femoris	1 sample per temperature and per time point (time points not specified)	α-actinin, calsequestrin 1, desmin, nebulin, titin, SERCA-1, SERCA-2, tropomyosin, cardiac troponin T (cTNT), laminin, µ-calpain	qualitative assessment of band presence/ absence over PMI	effect of temperature to degradation events (decrease in/loss of protein, degradation products) over time	increased protein degradation at higher temperatures
Jellinghaus et al., 2018 [20]	micro- organism	pig, bone	individuals not known, 16 bones (8 per group)	histology/ digital imaging, histology/ photometry	buried in boxes; 13–34 °C (monitored); 2 groups with different water infusion	0–3 months pm	right and left O. femoris	8 samples per time point (4)	collagen	quantitative assessment (software) of histological staining	effect of micro- organismic presence to collagenous to non-collagenous protein (Co/NCo) ratio	no significant effect of micro- organism presence
Jellinghaus et al., 2019 [21]	sex	human, bone	48 individuals	histology/digital imaging, histology/photometry	outdoor; cemetery and archeological samples (museum)	up to 171 years pm	O. femoris	48 samples at different time points	collagen	quantitative assessment (software) of histological staining	effect of gender to collagenous to non-collagenous protein (Co/Nco) ratio	decrease in ratio of Co/NCo concentration in males, increase in females
Kumar et al., 2016 [22]	temperature	human, heart	6 individuals	Western blot	12 °C, 20 ± 2 °C, 25 °C, 37 °C	unclear: probably up to 189 hpm	n.a.	not specified; several samples at several time points and temperatures	cTnT	not defined, probably percentage of intact protein	effect of temperature to degradation events (decrease in/loss of protein, degradation products) over time	increased protein degradation at higher temperatures
Kumar et al., 2016 [23]	cause of death	human, heart	50 individuals (10 per group)	Western blot	varying	unclear	n.a.	not specified; per group apparently different number of samples and different time points	cTnT	not defined, probably percentage of intact protein	effect of cause of death to degradation events (decrease in/loss of protein, degradation products) over time	dependence of protein degradation upon cause of death;
Pittner et al., 2016 [12]	age, body mass index, cause of death, sex, temperature (ADD)	human, skeletal muscle	40 individuals	casein zymography, Western blot	varying, accumulated degree days calculated	4–93 hpm	M. vastus lateralis	40 samples at different time points	desmin, calpain-1, calpain-2, cTnT, tropomyosin	presence and absence probability of bands at different accumulated degree days; correlation of band presence and absence with ADD	effect of age, BMI, sex and cause of death (COD) on timing and confidence intervals of degradation events	stronger correlation of degradation events with ADD in age and BMI corrected groups, no (major) effects by sex and COD
Pittner et al., 2020 [24]	body weight, exposure/environment, insect activity	pig, skeletal muscle	8 individuals (4 per group)	Western blot	outdoor; rectal and ambient temperature recorded	1–16 dpm	M. quadriceps femoris	8 samples (1 per animal/ 2 per group) per time point (10)	tropomyosin, desmin, vinculin, cTnT	quantitative (threshold) assessment of band presence/absence over PMI; temporal dependence of degradation events (changing probability of band presence over time)	effect of insect activity, body weight and exposure to protein degradation	loss of tissue hinders protein degradation analysis; robustness to body weight and exposure
Poloz and O’Day, 2009 [14]	temperature	mouse, lung and skeletal muscle	40 individuals	Western blot	laboratory- controlled; 5 °C, 10 °C, 21 °C	0–96 hpm	n.a.	4 samples per time point (4) and per temperature (3)	Calcineurin A (CnA), Myristoylated alanine-rich C-kinase substrate (MARCKS), Calcium/ calmodulin- dependent protein kinase II (CaMKII), Protein phosphatase 2A (PP2A)	band intensity, % of intact protein	effect of temperature to degradation (decrease in band intensity, degradation products) over time	increased protein degradation at higher temperatures
Wehner et al., 2006 [25]	temperature	human, brain and pancreas	500 individuals	immuno- histochemistry	varying	1–23 ± 1 dpm	frontal cortex, n.a.	number of samples per time point is unknown; 1 sample per tissue per individual	glial fibrillary acidic protein (GFAP), somatostatin	qualitative assessment of positive and negative immunostaining	effect of temperature (summer vs. winter seasons) to degradation events (presence and absence of staining)	faster decomposition in warmer season of the year

## Supplementary Materials

The following are available online at https://www.mdpi.com/article/10.3390/diagnostics11071146/s1. File S1: Instructions and questions to assess the risk of bias. File S2: Template to obtain the overall risk of bias. File S3: Assessment of the strength of the body of evidence. File S4: Summary of included studies. Table S1: Results of the assessment of the risk of bias for all included articles.
