# Supplementary material for: Influencing Factors on Postmortem Protein Degradation for PMI Estimation: A Systematic Review
Source: Diagnostics (Basel). 2021 Jun 23;11(7):1146. doi: 10.3390/diagnostics11071146 (PMC8304065; doi:10.3390/diagnostics11071146)
Supplement: Supplementary file 1 [file diagnostics-11-01146-s001.zip › File S4.pdf]

**Summary of individual studies**

Alibegović et al (2019) [1] investigated the degradation of cartilage in osteochondral specimens of human donors. Dissected samples were stored for up to 36 dpm at 11°C and at 35°C. Intensities of different classical histological collagen and proteoglycan stainings were assessed using a grading scale. Over the PMI of 36 days, a significant reduction in staining intensity was found at both temperatures, compared to the baseline samples. The effect of temperature, however, was not significant, indicating similar degradation rates in samples stored at 11°C and at 35°C in this experiment. Due to the small sample size and the qualitative assessment of data, the study was associated with a high risk of bias, although it was generally well designed in other aspects.

Foditsch et al. [2], used two pigs to investigate degradation of muscle proteins at 4°C (±1°) and at 22°C (±2°). To include the effect of temperature to decomposition, samples of *M. biceps femoris* were taken over a PMI of 21 days (cold environment) and 5 days (warm environment), respectively. Using SDS PAGE and Western blot analysis, advanced degradation was observed at 22°C for the following proteins: cardiac troponin T (cTnT), sarcoplasmic/endoplasmic reticulum calcium ATPase 1 (SERCA1), SERCA2, desmin, titin, and nebulin. Calsequestrin 1, laminin, tropomyosin and α-actinin remained stable over the investigated PMI. Due to its pilot character and small sample size, the work was assigned with a high risk of bias.

Jellinghaus et al. published two studies on postmortem degradation of collagen in 2018 [3] and 2019 [4]. Both studies tested two different methodological approaches. After differential histology staining of collagen and non-collagenous components of bone sections, the authors determined a collagen to non-collagen (Co/NCo) ratio by i) a photometric analysis of a destaining solution (method published by Boaks et al. [5]) and ii) a stereomicroscopic analysis using digital imaging data. In the first study [3], Jellinghaus and co-workers used samples of 16 porcine bones, buried in plastic boxes and stored under monitored conditions for 3 month. To investigate additional influence of microorganisms to collagen ratio changes, boxes with buried bones were flooded with hay infusions and compared to a control group (distilled water). The authors found a reduction of Co/NCo ratios over the investigated 3 month period, but no significant effect to collagen degradation by the presence of microorganisms. Although the study was generally well designed, the lack of reported effort to reduce bias in selecting a homogenous animal population resulted in a moderate risk of bias.

In the second study [4], the authors investigated human bones. Samples of 37 femoral bones were collected during exhumations (PMI 21-48 years) and autopsies, and samples of 11 femoral bones (PMI 135-153 years) were received from archaeological cases. Using the described methods, the authors reported a trend of increasing Co/nCo ratio if measured photometrically and a decreasing trend in measurements by stereomicroscopic image analysis. When sorted by gender, a sexual dimorphism was demonstrated. While the concentration of the Co/nCo ratio decreased in males (R=-0.62) as it would be expected, the ratio increased in females (R=0.24). The cause of this sexual dimorphism remains unclear but is discussed to arise from the high chronological age of the deceased females in

combination with diseases such as osteoporosis. Inaccuracies in describing the study design/outcome, as well as a lack in reporting sampling details, influencing factors and case data resulted in a high risk of bias rating.

Kumar et al. published two studies evaluating the influence of temperature [6] and cause of death [7] in 2016. In the first study, cardiac samples from 6 medico-legal autopsy cases were incubated at different temperatures (room temperature, 12°C, 25°C and 37°C) for different PMIs. In the second study, cardiac tissue samples of 6 groups of deceased individuals with different causes of death were analyzed including control, asphyxia, poisoning, burn, electrocution and myocardial infarction (n=10 in each group). Western blot analysis revealed that native cTnT degraded in a pseudo-linear relationship between percent of intact cTnT and PMI, and that cTnT is cleaved into several degradation products over time postmortem. Regarding the influence of temperature, the authors demonstrated increased degradation rates at higher temperatures. Regarding the effect of cause of death, an accelerated fragmentation rate of native cTnT is reported for cases in which causes of death were myocardial infarction. Data on exact incubation times and numbers of samples per storage temperature/cause of death-group are not provided. Both studies were rated with a high risk of bias because of small sample sizes, deficits in outcome reporting and (apparent) multiple use of original data.

Pittner et al. (2016) [8] used Western Blotting and casein zymography to investigate protein degradation in *M. vastus lateralis* samples from 40 human cases with known PMI [8]. To include the effect of temperature, the study employed accumulated degree days (ADD), a combined measure of PMI and environmental temperature, for the calculation of presence and absence probabilities of protein bands in specific time frames. While tropomyosin was present in all investigated cases regardless of ADD, cTnT, calpain and desmin showed distinct decomposition events (e.g. loss of native band, appearance of degradation products). The effects of age, body weight (as represented by BMI), cause of death, and sex were additionally tested using targeted cluster analysis. This was done in investigating an age-corrected group (excluding individuals below 18 and above 80 years), and a BMI corrected group (exclusion of individuals with a BMI below 19 and above 30). Results indicated stronger correlations between protein degradation and ADD and thus a potential effect of age and body weight. Cause of death and sex appeared to have no (major) effect on protein degradation dynamics, except for the ADD correlation of desmin degradation, which was slightly increased in females and decreased in males. The study was associated with a low risk of bias.

Pittner et al. (2020) [9] conducted a field study using 8 pig cadavers. To investigate influences of body mass as well as physical coverage and exposure, pigs with varying body weight were placed in different environments at a forensic research area in Germany. Among two other methods (morphology, entomology), postmortem protein degradation of skeletal muscle tissue (*M. biceps femoris*) was analyzed by Western blots over a PMI of 14 days. Analyzed proteins (tropomyosin, cTnT, desmin and vinculin) depicted typical decomposition pattern, including a loss of native bands

and the appearance of degradation products, in correlation with the PMI. The authors concluded that protein degradation was largely robust towards the influencing factors “body weight” and “exposure”. Due to the small sample size, the study was associated with a high risk of bias.

Poloz and O’Day (2009) [10], also using Western blotting, investigated lung and skeletal muscle in mice. To test the effect of temperature, the animals were stored at 5 °C, 10 °C and 21 °C and sampled at four time points within 96 hpm. In both tissues, most tested proteins (Calcineurin A (CnA), calmodulin dependent kinase II (CaMKII), myristoylated alanine-rich C-kinase substrate (MARCKs), protein phosphatase 2A (PP2A) were found to degrade postmortem, showing significant effects of the PMI on protein degradation. The degradation of CnA and PP2A, in particular, wer
e significantly affected by temperature, with the most drastic breakdown at the highest temperature of 21°C. Although the study is generally well designed, details about the sampling procedure and the sampling sites are lacking, which resulted in a judgement of moderate risk of bias.

In 2006, Wehner et al. [11] investigated the proteins glial fibrillary acidic protein (GFAP) and somatostatin in human brain and pancreas, respectively. Using immunohistochemistry, stainability of GFAP and somatostatin were evaluated in samples of 500 individuals with varying time of death (PMI between 1 and 23 days). GFAP staining persisted until day 3 postmortem, and no staining was consistently found after day 14; somatostatin was constantly detected between 1 and 2 days postmortem, but its stainability was consistently lost at PMIs of 11 days or longer. In relation to temperature effects, authors found that in corpses with similar PMI, more negative immunoreactions are seen in warmer months of the year compared to colder months. Thus, a seasonal temperature dependence of degradation is indicated with decomposition being faster at high temperatures and slower at low temperatures. Although the study was based on a large number of human cases, it had to be associated with an overall high risk of bias due to the lack of a detailed description of measurement procedures and data analysis.

**References**


1. Alibegović, A.; Blagus, R.; Martinez, I.Z. Safranin O without Fast Green Is the Best Staining Method for Testing the Degradation of Macromolecules in a Cartilage Extracellular Matrix for the Determination of the Postmortem Interval. *Forensic Sci. Med. Pathol.* **2019**, doi:10.1007/s12024-019-00208-0.
2. Foditsch, E.E.; Saenger, A.M.; Monticelli, F.C. Skeletal Muscle Proteins: A New Approach to Delimitate the Time since Death. *Int. J. Legal Med.* **2016**, *130*, 433–440, doi:10.1007/s00414-015-1204-4.
3. Jellinghaus, K.; Hachmann, C.; Höland, K.; Bohnert, M.; Wittwer-Backofen, U. Collagen Degradation as a Possibility to Determine the Post-Mortem Interval (PMI) of Animal Bones: A Validation Study Referring to an Original Study of Boaks et al. (2014). *Int. J. Legal Med.* **2018**, *132*, 753–763, doi:10.1007/s00414-017-1747-7.
4. Jellinghaus, K.; Urban, P.K.; Hachmann, C.; Bohnert, M.; Hotz, G.; Rosendahl, W.; Wittwer-Backofen, U. Collagen Degradation as a Possibility to Determine the Post-Mortem Interval (PMI) of Human Bones in a Forensic Context – A Survey. *Leg. Med.* **2019**, *36*, 96–102, doi:10.1016/j.legalmed.2018.11.009.

5. Boaks, A.; Siwek, D.; Mortazavi, F. The Temporal Degradation of Bone Collagen: A Histochemical Approach. *Forensic Sci. Int.* **2014**, *240*, 104–110, doi:10.1016/j.forsciint.2014.04.008.
6. Kumar, S.; Ali, W.; Singh, U.S.; Kumar, A.; Bhattacharya, S.; Verma, A.K.; Rupani, R. Temperature-Dependent Postmortem Changes in Human Cardiac Troponin-T (CTnT): An Approach in Estimation of Time Since Death. *J. Forensic Sci.* **2016**, *61*, S241–S245, doi:10.1111/1556-4029.12928.
7. Kumar, S.; Ali, W.; Bhattacharya, S.; Verma, A.K. The Effect of Elapsed Time on Cardiac Troponin-T (CTnT) Degradation and Its Dependency on the Cause of Death. *J. Forensic Leg. Med.* **2016**, *40*, 16–21, doi:10.1016/j.jflm.2016.02.002.
8. Pittner, S.; Ehrenfellner, B.; Monticelli, F.C.; Zissler, A.; Sänger, A.M.; Stoiber, W.; Steinbacher, P. Postmortem Muscle Protein Degradation in Humans as a Tool for PMI Delimitation. *Int. J. Legal Med.* **2016**, doi:10.1007/s00414-016-1349-9.
9. Pittner, S.; Bugelli, V.; Weitgasser, K.; Zissler, A.; Sanit, S.; Lutz, L.; Monticelli, F.; Campobasso, C.P.; Steinbacher, P.; Amendt, J. A Field Study to Evaluate PMI Estimation Methods for Advanced Decomposition Stages. *Int. J. Legal Med.* **2020**, doi:10.1007/s00414-020-02278-0.
10. Poloz, Y.O.; O’Day, D.H. Determining Time of Death: Temperature-Dependent Postmortem Changes in Calcineurin A, MARCKS, CaMKII, and Protein Phosphatase 2A in Mouse. *Int. J. Legal Med.* **2009**, *123*, 305–314, doi:10.1007/s00414-009-0343-x.
11. Wehner, F.; Steinriede, A.; Martin, D.; Wehner, H.-D. Two-Tailed Delimitation of the Time of Death by Immunohistochemical Detection of Somatostatin and GFAP. *Forensic Sci. Med. Pathol.* **2006**, *2*, 241–247, doi:10.1385/FSMP:2:4:241.
