# Supplementary material for: Influencing Factors on Postmortem Protein Degradation for PMI Estimation: A Systematic Review
Source: Diagnostics (Basel). 2021 Jun 23;11(7):1146. doi: 10.3390/diagnostics11071146 (PMC8304065; doi:10.3390/diagnostics11071146)
Supplement: Supplementary file 1 [file diagnostics-11-01146-s001.zip › File S1.pdf]

Assessment of risk of bias

The review authors developed a tool that provides a framework for considering the potential risk of bias in the specific studies included in this review. The structure and wording of this risk of bias tool is adapted from the Cochrane RoB 2.0 tool designed for Randomized Clinical Trials [1, 2]. The authors very much agree on the importance to assess the risk of bias, also in studies within non-clinical settings. The tool represents a mixture of novel aspects and already approved domains of the Cochrane Collaboration.

The assessment is structured in a series of domains through which bias might be introduced into a research study that is reported in conventional form including material and methods and results sections. We applied the assessment to (biomedical) original research and case studies using animal and human samples.

The tool contained the following 6 domains of bias:

1. Bias arising from study design
2. Bias due to inaccuracy in reporting the methods
3. Bias due to missing outcome data
4. Bias in measurement of the outcome
5. Bias in selection of the reported results
6. Bias arising from multiple use of data

For each domain the authors defined specific signaling questions and/or included/adapted existing questions originating from the RoB 2.0 tool. The purpose of these questions was to facilitate judgements about the risk of bias for each domain. The questions were phrased such that a response like "yes" is indicative for a certain judgement of risk of bias (low, medium or high risk of bias). Some questions had to be answered only if the answer to a previous question was "yes" or "probably yes" (and "no", "probably no", "no information").

Response options to signaling questions were the following:

1. Y: Yes
2. PY: Probably yes
3. N: No
4. PN: Probably no
5. NI: No information

When using “probably” it is implied that a judgment has been made by the reviewer. The “no information” response was avoided if possible and used only when insufficient data were reported permitting a reasonable judgement to be made. A specific response to a question did not affect answers to other questions within the same, or other domains.

Similar to the RoB 2.0 tool, judgements were made for each domain (“domain-level judgement”) and for the sum of all domains (“overall risk of bias”). Keys used for domain-level judgements and for overall risk of bias are presented in the supplementary material file S2.

Following judgment options were chosen:

1. Low risk of bias
2. Moderate risk of bias
3. High risk of bias

Signaling questions: information and explanation

1.1 Was the sample size chosen adequately (to appropriately address the research hypothesis)? (Y/PY/N/PN/NI)

The “sample size” was defined as the number of individuals, or the number of individuals actually used to generate data. Note that an appropriate number of individuals generally depends on the context of the respective study. The sample size is adequately chosen, in case the obtained data appropriately reflected the studied population in both, the number of individuals and the range of variance of the target variable (e.g. time points postmortem). Generally, a large range of variance of the target variable requires large sample sizes.
For animal studies using a homogenous group of animals the authors agreed that a minimum of 5 individuals per group (e.g. time point) were adequate (=enough to be confident with the findings) within the included studies (judgment “Yes”). A sample size of 4 to 3 individuals per group was judged with “Probably yes”. A sample size below 3 individuals was considered insufficient and therefore judged with “No”.

1.2.1 <u>If Y/PY to 1.1</u> Were appropriate control samples used that serve as reference/comparative values within the outcome analysis domain?? (Y/ N/NI)
The question was answered with “Yes” if appropriate control (“zero-hour”) samples obtained at the time of death (especially relevant for animal studies) or multiple samples at different time points postmortem (especially relevant for human studies) were used to serve as comparative values.

1.2.2 <u>If Y to 1.2.1</u> Was there any effort to reduce risk of bias in selecting an appropriate population fitting into the context of the study? (Y/PY/N/PN/NI)

Especially to estimate influencing factors, the study conductor should be aware of the patients' and animals' histories and data. E.g.: In case of using myocard samples for PMI estimation based on protein degradation, the study conductor must consider and report about the antemortem clinical history, cause of death, etc. and eventually exclude the patient/case from study participation (e.g. death by myocardial infarction, existing cardiomyopathy or other diseases which potentially affects protein degradation) and generalization of the results should be avoided, respectively.

"Yes" was assigned, if study conductors considered and/or reported about data of patients/cases (regarding age, sex, BMI) and appropriate inclusion and exclusion criteria and/or other attempts to reduce the risk of bias in this issue. "Yes" is also assigned, if study conductors used standardized, healthy animals and/or animals whose manner of death was considered/reported and/or if other attempts were made to reduce the risk of bias on this behalf. The animal group had to be selected/reported as homogenous as possible (e.g.: male adult Sprague Dawley rats).

2.1 Was the study design and outcome reported consistently and unambiguously? (Y/N)

The reader/reviewer must be able to follow the content and structure of the study. The respective results must be clearly stated. If this was the case, the question was answered with "Yes".

2.2 Was the sampling (procedure and site) reported comprehensibly? (Y/N)

The question was answered with "Yes", when the sampling procedure was clearly reported (e.g. muscle tissue pieces (100mg) were dissected from the belly of the *M. biceps femoris* in a depth of 1 cm). This is important to guarantee reproducibility. An attempt in reporting the sampling site with (e.g.) "skeletal muscle" was judged worse than e.g. "thyroid gland". This was done because the thyroid gland is a homogenous organ within a specified mammalian body part, whereas skeletal muscle is spread over the whole body and specifications of the individual muscle used are necessary to reproduce the sampling procedure.

2.3 Were influencing factors and/or environmental conditions reported that possibly influence the outcome domain? (Y/N)

In case of animal studies, storage conditions of samples/cadavers had to be clearly reported (temperature, humidity, etc.) to answer this question with "Yes". Conductors of studies using human tissue should comment on environmental conditions and/or storage conditions of corpses after death/discovery. The authors are aware that exact environmental conditions are unknown in many cases. It is the study conductors' responsibility to define appropriate context-specific inclusion and/or exclusion criteria (e.g. inclusion when PMI is defined, exclusion when PMI is unknown).

2.4 Were methodical steps reported comprehensively and traceable from sample preparation to data acquisition? (Y/N)

The question was answered with “Yes”, when the reader/reviewer is able to comprehend all steps between the dissections of samples to the generation of results ready for analyses. Methods had to be described in detail to guarantee reproducibility.

2.5 Were details reported regarding the measurement procedure and data analysis that are essential for the outcome domain (endpoint of interest)? (Y/N)

"Yes" was assigned, when measurement procedures, data acquisition and analyses are reported in such detail that replication experiments and respective data validation and comparison is possible.

3.1. Were outcome data available for all, or nearly all, samples used? (Y/PY/N/PN/NI)

Especially in studies on humans it is not unusual that individuals, tissues, or samples drop out of a study at some point during the course of experimentation, or that specific data has to be excluded. This is per se not an indication for an increased risk of bias. The following questions aim to assess the according risk. "Yes" was assigned when data was available/reported for all samples used.

3.2.1. If N/PN/NI to 3.1 Are the proportions of missing outcome data similar across study groups? (Y/PY/N/PN/NI)

For statistic analysis it is important to retain similar and sufficient sample sizes. Excluded data towards a single, or several specific study group/s might indicate correlations and eventually inappropriate methodology. The question was answered with "Yes", when excluded data are equally/similarly distributed across study groups.

3.2.2. If N/PN/NI to 3.1 Are the reasons for (partially) missing outcome data reported? (Y/N)

When appropriately handled and accordingly discussed, the risk of bias due to reported missing outcome data could be reduced. "Yes" was assigned if a clear, comprehensive explanation is presented and data is discussed with caution.

3.2.3. If N/PN/NI to 3.1 Is there evidence that results were robust to the presence of missing outcome data? (Y/NI/N)

Especially with study designs with considerably large sample sizes, a partial lack of outcome data can be relativized. "Yes" was answered if there is substantial evidence, that the study outcome is unaffected by missing data.

4.1. Were outcome assessors aware of the study group/time point/individual sample data? (Y/PY/NI/N/PN)

The question was answered "No" when samples and/or data were reportedly anonymized, and/or other efforts are reported to effectively blind the outcome assessors. Please see also [2].

4.2. If Y/PY/NI to 4.1 Was the assessment of the outcome likely to be influenced by knowledge of the study group/time point/individual sample data? (Y/PY/N/PN)

Knowledge of the assigned experimental group/intervention may impact the outcome. The importance of assessor blinding is not limited to clinical research. The authors, however, agreed that blinding can be of different importance depending on the evaluation method used. Thus, assessor blinding is especially important in case of subjective methods (e.g. subjective grading of histological changes by microscopy) and diminishes in importance by applying objective, computer-assisted analysis (threshold is calculated from intensity measurements). Please see also [2].

5.1 Are the reported outcome data likely to have been selected from multiple outcome measurements (methods, time points, conditions, etc.)? (Y/PY/N/PN/NI)

In order to assess the reliability and reproducibility of a study outcome, a gapless study design, documentation and reporting is required. Indication for outcome-motivated experimentation and/or biased study design (e.g. intentional omission of samples) is problematic for meta-analysis. "Yes" was answered when multiple measurements were made, but only a subset is reported on the basis of the results (e.g. statistical significance).

5.2 Are the reported outcome data likely to have been selected from multiple analyses of the data (values, percentages, ratios)? (Y/PY/N/PN/NI)

To be able to assess the reliability and impact of a study outcome, it is required that the entirety of data is made available. A selected reporting of "positive" outcome data can bear bias for meta-analysis. The question was answered with "Yes" when multiple datasets are generated but only a subset is reported on the basis of the results (e.g. statistical significance).

6.1 Is there evidence that the same ***individuals*** *were used in other included studies, published earlier? (Y/PY/N/PN)*

It is often reasonable to use individuals several times for different research approaches. Be it to keep the sample size of the used animals as small as possible or to sample / use human tissue samples several times. Generally, this is not problematic as it does not influence the quality of the studies per se. Nevertheless, a meta-analysis can be affected in certain circumstances if samples come from the same individual (e.g. samples from different tissues, published in two or more articles, but originating

from the same case/s). For this reason the authors agreed that using individuals several times entails moderate risk of bias.

6.2.1 Is there evidence that the same ***sample*** *material was used in other included studies, published earlier? (Y/PY/N/PN)*
It can be reasonable to use the same samples of one individual for different research approaches (e.g. different analysis methods). Generally, this doesn't influence the quality of the studies per se. However, a meta-analysis is certainly affected if the same samples were used in two or more articles. For this reason the authors agreed that using samples several times entails moderate risk of bias. Under specific circumstances (see 6.2.1) a high risk of bias was assessed.

6.2.2 <u>If Y/PY to 6.2.1</u> Is the multiple use of samples likely to affect meta-analysis? (Y/PY/N/PN)
The question was answered with "No", when the same samples were investigated in two or more studies, but different analysis methods were used (e.g. one article on western blotting, one article on mass spectrometry), or if different markers (e.g. different proteins) were investigated. "Yes" was answered, when experiments from a previous work were replicated using the same samples.

6.3 Is there evidence that the same ***data*** *were used in other included studies published earlier? (Y/PY/N/PN)*
It entails a high risk of bias for meta-analysis if the same data are published in two or more articles, regardless if there is an explanation proposed, or not.

References

1. Higgins J, Sterne J, Savović J, et al (2016) A revised tool for assessing risk of bias in randomized trials In: Chandler J, McKenzie J, Boutron I, Welch V (editors). Cochrane Methods. Cochrane Database Syst Rev Issue 10 (Suppl 1): https://doi.org/dx.doi.org/10.1002/14651858.CD201601.

2. Higgins J, Savović J, Page M, Sterne J (2016) Revised Cochrane risk of bias tool for randomized trials (RoB 2.0)
