# Supplementary material for: Influencing Factors on Postmortem Protein Degradation for PMI Estimation: A Systematic Review
Source: Diagnostics (Basel). 2021 Jun 23;11(7):1146. doi: 10.3390/diagnostics11071146 (PMC8304065; doi:10.3390/diagnostics11071146)
Supplement: Supplementary file 1 [file diagnostics-11-01146-s001.zip › Table S1.pdf]

| Domain | Question | Alibegovic et al. 2019 [18] | Foditsch et al. 2016 [19] | Jellinghaus et al. 2018 [20] | Jellinghaus et al. 2019 [21] | Kumar et al. 2016 [22] | Kumar et al. 2016 [23] | Pittner et al. 2016 [12] | Pittner et al. 2020 [24] | Poloz and O'Day 2009 [14] | Wehner et al. 2006 [25] |
|---|---|---|---|---|---|---|---|---|---|---|---|
| Bias arising from study design | 1.1 Was the sample size chosen adequately (to appropriately address the research hypothesis)? (Y/PY/N/PN/NI) | N | N | Y | Y | N | Y | Y | N | PY | Y |
| | 1.2.1 If Y/PY to 1.1 Were appropriate control samples used that serve as references/comparative values within the outcome analysis domain? (Y/PY/N/PN/NI) | | | Y | Y | | PY | Y | | Y | Y |
| | 1.2.2 If Y/PY to 1.2.1 Was there any effort to reduce risk of bias in selecting an appropriate population fitting into the context of the study? (Y/PY/N/PN/NI) | | | N | N | | PY | Y | | PY | Y |
| | **Risk of bias judgement** | high | high | moderate | moderate | high | low | low | high | low | low |
| Bias due to imprecise reporting | 2.1 Was the study design and outcome reported consistently and unambigously? | Y | Y | Y | N | N | Y | Y | Y | Y | Y |
| | 2.2 Was the sampling (procedure and site) reported comprehensibly? (Y/N) | Y | Y | Y | N | N | N | Y | Y | N | Y |
| | 2.3 Were influencing factors and/or environmental conditions reported that possibly influence the outcome domain? (Y/N) | Y | Y | Y | N | Y | N | Y | Y | Y | Y |
| | 2.4 Were methodical steps reported comprehensively and traceable from sample preparation to data acquisition? (Y/N) | Y | Y | Y | Y | Y | Y | Y | Y | Y | N |
| | 2.5 Were details reported regarding the measurement procedure and data analysis that are essential for the outcome domain (endpoint of interest)? (Y/N) | Y | N | Y | Y | N | N | Y | Y | Y | N |
| | **Risk of bias judgement** | low | moderate | low | high | moderate | high | low | low | moderate | high |
| Bias due to missing outcome data | 3.1. Were outcome data available for all, or nearly all, samples used? (Y/PY/N/PN/NI) | Y | PY | Y | PY | N | N | Y | N | Y | PN |
| | 3.2.1. If N/PN/NI to 3.1 Are the proportions of missing outcome data similar across study groups? (Y/PY/N/PN/NI) | | | | | NI | Y | | Y | | N |
| | 3.2.2. If N/PN/NI to 3.1 Are the reasons for (partially) missing outcome data reported? (Y/N) | | | | | N | N | | Y | | N |
| | 3.2.3. If N/PN/NI to 3.1 Is there evidence that results were robust to the presence of missing outcome data? (Y/NI/N) | | | | | N | NI | | NI | | NI |
| | **Risk of bias judgement** | low | low | low | low | high | high | low | moderate | low | high |
| Bias in measurement of the outcome | 4.1. Were outcome assessors aware of the study group/time point/individual sample data? (Y/PY/NI/N/PN) | PN | NI | NI | N | NI | NI | NI | NI | NI | NI |
| | 4.2. If Y/PY/NI to 4.1 Was the assessment of the outcome likely to be influenced by knowledge of the study group/time point/individual sample data? (Y/PY/N/PN) | PN | PY | PN | | PN | PN | PN | PN | PN | PY |
| | **Risk of bias judgement** | low | moderate | low | low | low | low | low | low | low | moderate |
| Bias in selection of the reported result | 5.1 Are the reported outcome data likely to have been selected from multiple outcome measurements (methods, time points, conditions, etc.)? (Y/PY/N/PN/NI) | N | PN | N | PN | PY | PY | N | N | N | N |
| | 5.2 Are the reported outcome data likely to have been selected from multiple analyses of the data (values, percentages, ratios)? (Y/PY/N/PN/NI) | N | N | N | N | N | N | N | N | N | N |
| | **Risk of bias judgement** | low | low | low | low | moderate | moderate | low | low | low | low |
| Bias arising from multiple use of data | 6.1 Is there evidence that the same **individuals** were used in other included studies, published earlier? (Y/PY/N/PN) | N | N | N | N | N | Y | N | N | N | PY |
| | 6.2.1 If Y/PY to 6.1 Is there evidence that the same **sample** material was used in other included studies, published earlier? (Y/PY/N/PN) | | | | | | Y | | | | PY |
| | 6.2.2 If Y/PY to 6.2.1 Is the multiple use of samples likley to affect meta-analysis? (Y/PY/N/PN) | | | | | | N | | | | N |
| | 6.3 If Y/PY to 6.2.1 Is there evidence that the same **data** were used in other included studies, published earlier? (Y/PY/N/PN) | | | | | | Y* | | | | N |
| | **Risk of bias judgement** | low | low | low | low | low | low* | low | low | low | moderate |
| Overall bias | **Risk of bias judgement** | high | high | moderate | high | high | high | low | high | moderate | high |

*although evidence exists that the same data were used in other included studies, meta analyses are not affected in this particular study. Because in Kumar et al. 2016 [ ] and Kumar et al. 2016 [ ] different influencing factors were investigated (temperature and cause of death), data are incorporated in separated meta-analyses.
